# The Process of Developing an Assessment Checklist for Simulated Infant Respiratory Distress Using a Modified Delphi Method: A Mixed Methods Study

**DOI:** 10.7759/cureus.7866

**Published:** 2020-04-28

**Authors:** Justin M Jeffers, William Golden, Amit K Pahwa, Stacy Cooper, David Cooke, Rebekah Reisig, Christopher Grybauskas, Eric Balighian, Emily Frosch, John H Shatzer

**Affiliations:** 1 Pediatrics, The Johns Hopkins University School of Medicine, Baltimore, USA; 2 Pediatrics, Johns Hopkins University, Baltimore, USA; 3 Medicine, Pediatrics, Johns Hopkins University, Baltimore, USA; 4 Psychiatry, Johns Hopkins University, Baltimore, USA; 5 School of Education, Johns Hopkins University, Baltimore, USA; 6 Medical Informatics, The Johns Hopkins University School of Medicine, Baltimore, USA

**Keywords:** delphi method, assessment, checklist development, simulation-based learning, respiratory distress, medical education, simulation in medical education, quantitative and mixed methods research

## Abstract

Introduction

Assessing clinical performance, such as managing respiratory distress, in clinical trainees is challenging yet important. Our objective was to describe and evaluate an integrative and iterative approach to developing a checklist measuring simulated clinical performance for infant respiratory distress.

Methods

We implemented a five-step modified Delphi process with an embedded qualitative component. An implementation period occurred followed by a second qualitative data collection. Validity evidence was collected throughout the process.

Results

A 19-item assessment checklist was developed for managing infant respiratory distress by medical student learners in a simulation-based setting. The iterative process provided content validity while the qualitative data provided response process validity. Cohen kappa was 0.82 indicating strong rater agreement. The assessment checklist was found to be easy to use and measure what was intended.

Conclusion

We developed an accurate and reliable assessment checklist for medical student learners in a simulation-based learning setting with high interrater reliability and validity evidence. Given its ease of use, we encourage medical educators and researchers to utilize this method to develop and implement assessment checklists for their interventions.

## Introduction

Assessing clinical performance in medicine is important for many reasons, as it allows educators to determine performance gaps, identify strengths and weaknesses, and perform needs assessments for future educational interventions [1]. However, clinical performance is a complex entity and is often viewed as having both a process and an outcome component [2]. In clinical practice, outcomes such as procedural success or performance of hand washing by clinicians are more easily measured objectively. In contrast, process measurement relates to what a person or team does in a situation. This parameter is more challenging to measure, but can be assessed either subjectively and/or objectively [1]. While subjective measures rely largely on expert observation, checklists can provide accurate and reliable information regarding performance.

A classic checklist uses dichotomous items such as done/not done. This type of checklist can be effective for procedural tasks but may not be robust enough to evaluate clinical performance [3]. For complex clinical tasks, extra layers to a checklist are required, such as additional categories (done/done incorrectly/not done) and weighted items based on importance [4-6]. Including these extra layers helps create a more refined and accurate checklist [7].

The aims of this study were to 1) develop a checklist for assessing the clinical performance of managing infant respiratory distress and evaluate validity evidence, 2) qualitatively investigate the development process via questionnaire and focus group, and 3) qualitatively investigate the functionality, response process validity, and ease of use of the developed checklist.

## Materials and methods

This project was an embedded and sequential mixed methods study evaluating a checklist to measure pediatric clerkship student performance of managing a simulated infant respiratory distress scenario and the process used in its development. It followed a quantitative (QUANT) -> qualitative (QUAL) -> QUANT -> QUAL structure, and used a modified Delphi method based on the work of Schmutz et al. [7].

We developed and tested the assessment checklist from April through December 2018. Participants in the study consented to participate and to be videotaped.

Subjects

Purposeful homogenous sampling was done to select the expert panel for the checklist development process. The panel selected have expertise in infant respiratory distress and include pediatric faculty in emergency medicine, oncology, neonatology, and hospital medicine.

The participants of the checklist itself are a probabilistic convenience sample of pediatric clerkship medical students (third- and fourth-year students). During their clerkship, students participate in a curriculum for obtaining and practicing pediatric knowledge and skills called PRECEDE (PRE-Clerkship EDucational Exercises) [8-10]. One module during PRECEDE is a one-hour simulation-based learning (SBL) curriculum for assessing and managing infant respiratory distress due to bronchiolitis. These modules were video recorded for this study.

Five-step development process

The primary author developed an initial draft of a checklist (Step 1) for a simulated scenario of infant respiratory distress based on published guidelines and clinical experience [11]. The initial draft was sent via e-mail to the other five members of the expert panel for review using a modified Delphi process (Step 2) [12,13]. The panel was encouraged to offer suggestions and edits within one week. The primary author integrated these suggestions and edits and redistributed the checklist for the next round of suggestions and edits. This process was completed once unanimous consensus was achieved.

Two content experts (PI plus one person not associate with the development process) piloted the consensus checklist to improve the accuracy of the tool (Step 3). Ten video-recorded scenarios of management of simulated infant respiratory distress by groups of 3-5 students on their pediatric clerkship were reviewed. The same 10 scenarios were used for interrater reliability testing. The original six-person panel reviewed the post-pilot checklist via e-mail (Step 4). Suggested changes were discussed and agreed upon by the panel.

An additional four pediatric emergency medicine experts were recruited via volunteer e-mail request to assist with item weighting (Step 5). This process aimed to place greater importance on certain items and avoid excessive penalization for missing a less important item.

After checklist development was completed, an implementation trial was done to assess ease of use and response process validity. Faculty for the PRECEDE module were asked to use the checklist in real-time twice. The final checklist is shown in Table 1.

**Table 1:** Final Infant Respiratory Distress Checklist ECG: Electrocardiogram; SpO2: Oxygen saturation; BP: Blood pressure; secs: seconds; NRB: Non-rebreather; BMV: Bag mask ventilation; CRT: Capillary refill time; L: liter; O2: Oxygen.

Stage of Care (time in minutes)	Item no.	Item	Not Done (0 points)	Partially or Incorrectly Done (1 point)	Done correctly, and completely (2 points)	Weighting	Item Score
Situational Awareness/General Tasks (0-2) Objectives: 1, 2	1.1	Turns on Lights		Done but took longer than 5 secs	Done within 5 secs	4	
	1.2	Lowers bed rails		Done but longer than 30 secs	Done within 30 secs	3	
	1.3	Removes patient from car seat		Done but longer than 60 secs	Done within 60 secs	4.5	
	1.4	Removes patient gown		Done but longer than 90 secs	Done within 90 secs	4	
	1.5	Gathers brief but appropriate history		Required prompting, or inappropriate details	Appropriate and complete information gathered	4.5	
	1.6	Applies appropriate personal protection equipment (gloves for patient contact, mask if near airway)		Some but not all apply personal protection equipment	All apply equipment within 60 secs	3.5	
	1.7	Place patient on monitor (ECG, Sp02, BP)		Done but longer than 90 secs	Done within 90 secs	5	
	1.8	Clear and defined role assignment (leader, airway x 2, primary assessor, family liaison)		Roles differentiated but not clearly assigned OR 3 or fewer roles assigned	4 or more clearly assigned and defined roles	4	
Initial Management (0-3) Objectives: 1, 2, 3, 4							
	2.1	Assess airway and breathing via clear effort such as auscultation, verbal recognition of respiratory vital signs, etc.		Assess one or the other, or not timely	Done within 30 secs	5	
	2.2	Recognizes respiratory distress via verbalization or clear attempt at intervention		Done but longer than 90 secs	Done within 90 secs	5	
	2.3	Attempts airway opening maneuvers – Head tilt, jaw thrust, chin lift, or shoulder roll		Only does 1 or multiple but incorrectly done, or not timely	Does multiple correctly within 90 secs	5	
	2.4	Apply O2		Nasal cannula > 6L or NRB <10L or not timely.	100% NRB at >10L within 120 secs or escalated approach within 180 secs	5	
	2.5	Assesses circulation – HR, BP, access, CRT		Done but not all measures or longer than 120 secs	All measures done within 120 secs	5	
	2.6	Utilizes appropriate team-based communication – closed loop within team, appropriate and timely family communication, frequent verbal reassessment/summary		Rarely or sometimes	Usually or always	4	
Escalation of Care (2-10) Objectives: 1, 2, 3, 4							
	3.1	Recognizes initial interventions are not working		Either verbalize OR intervene OR >30 secs from placing O2	Verbalize AND intervene within 30 secs of placing O2	4.5	
	3.2	Places oral and/or nasal airway		Placed but did not measure for size OR longer than 90 secs	Done with proper size AND within 60 secs	3.5	
	3.3	Initiates BMV using proper technique (EC or two-person method)		Done but improper technique OR longer than 60 secs	Done properly within 60 secs of recognizing need for further intervention	4.5	
	3.4	Calls for more help			Verbalized at any point during scenario	4.5	
	3.5	Reassess after each intervention		Reassess after 2 or fewer interventions	Reassess after 3 or greater interventions	4.5	

Data collection and analysis

Numerous forms of validity evidence were evaluated including content, construct, internal consistency, and response process. Content validity was performed via the iterative process itself [1]. Construct validity was analyzed via the pilot portion of checklist development. Inter-rater reliability (as measured by Cohen’s kappa) provided data for internal consistency. Cohen’s kappa was calculated for each checklist section as well as the overall checklist.

Qualitative data collection occurred twice. After the checklist was developed, a brief anonymous on-line survey was distributed to the panel considering thoughts and perceptions about the process. In particular, they were asked to compare their experience to other checklist development processes they may have participated in previously. After the small implementation trial, qualitative data was collected via similar means investigating checklist ease of use and response process validity. Questions were based on a five-point scale. Means and standard deviations (SD) were calculated.

Individual item weights were obtained by e-mail. Ten participants were asked to provide each checklist item a rank from one (not important) to five (essential). Mean scores were calculated for each item along with SD. Quantitative analysis was performed via Stata/SE 15.1 for Windows (64-bit x86-64), (Statacorp LLC, College Station, TX).

## Results

A total of 90 students consented to participate in this study. Consent included study participation as well as to video and audio recording.

Step 1: Initial draft checklist development

The primary author developed a checklist consisting of three categories: (1) Situational Awareness/General Tasks, (2) Initial Management, and (3) Escalation of Care with 17 potential items total. Each checklist item had three possible outcomes: (1) Done completely and correctly (2 points), (2) Partially or incorrectly done (1 point), (3) Not done (0 points). Each item included written descriptive anchors. This step required approximately six hours.

Step 2: Delphi review rounds

Six experts in pediatrics (all are authors from this institution) participated in the modified Delphi review rounds. Any suggestions made during each round were distributed to the group for consensus. Changes made to the checklist required unanimous agreement. During the first review round, two items were added, and three items were edited (all related to time to completion of a certain event). The second round resulted in unanimous agreement with the first-round changes. During the third round, the experts recognized a need to link the checklist to the known objectives for the educational intervention being assessed. The four objectives were reviewed, updated, and added to the checklist. The (final) fourth round noted unanimous agreement, and no further edits were suggested.

The resultant checklist consisted of 19 items across three categories. Category 1 (situation awareness/general tasks) had eight items and related to objectives one and two. Category 2 (initial management) had six items and related to all four objectives. Category 3 (escalation of care) had five items and related to all four objectives. Each round required approximately 45-60 minutes.

Step 3: Pilot testing

Using the checklist, the primary author and a pediatric emergency medicine expert (not involved with the checklist development process) individually rated 10 videos recorded SBL scenarios of infant respiratory distress. The purpose of this step was to identify items that were challenging to score, needed further clarification or more specificity, and to determine if the tool assesses what it was meant to assess (construct validity). No items were added. The time component of two items was increased (reviewers noticed the time allotted was not a reasonable or realistic amount of time for task completion). This step required approximately six hours per reviewer.

Step 4: Final modified Delphi round

After piloting, all six experts agreed with the final version of the checklist.

Step 5: Item weighing, internal consistency, and validity

Mean weighted scores ranged from three to five and were rounded to the nearest half integer. Four of the 19 items had a SD of greater than one, with three of those items (“applying appropriate personal protection equipment”, “gathers brief but appropriate history”, and “lowers bed rails”) occurring in the first section of the checklist. The fourth item with a SD greater than one was in the final section: “places oral/nasal airway”. Of the six items in the second section, “Initial Management”, five had a SD of zero. This step required approximately 30-45 minutes to complete.

Interrater reliability for the checklist was k = 0.82 (Table 2). The thorough, iterative checklist development process used to derive items provided content validity [1].

**Table 2:** Interrater Reliability as Measured by Cohen's Kappa

Section	Cohen's k (95% CI)
Situational Awareness/General Tasks	0.84 (0.77-0.92)
Initial Management	0.79 (0.70-0.89)
Escalation of Care	0.81 (0.70-0.91)
Overall	0.82 (0.77-0.87)

Qualitative results

All six checklist development participants responded to the survey questions. Three participants reported previous participation in an assessment tool development process and stated that our process required less time than their previous experiences. All participants found the process to be easier than they expected and reported each round of revisions took less time than they expected. Participants noted a willingness to participate in the process again.

In support of response process validity, all participants felt the checklist appropriately and accurately measured the stated objectives (4.7/5, SD 0.52) and performance (4.7/5, SD 0.52) for this learner group. Most found the checklist easy to use (4.1/5, SD 1.12). One of the participants found it challenging to score the checklist in real-time while also operating the simulator. Representative quotations from faculty are noted in Table 3.

**Table 3:** Sample Quotations from Qualitative Data Collection

Question	Response
Please describe your thoughts on the time needed for this assessment checklist development process.	“I was expecting it to take much longer than it did. I spent no more than 20 minutes on each review.” “I was initially hesitant to participate due to the time commitment, but it wasn’t as bad as I anticipated.”
How did this assessment checklist development process compare to others you have experienced?	“I appreciated being able to complete on my own time.” “This process took much less time than the other checklist I helped develop.”
Would you participate in this assessment checklist development process again?	“Yes.” “Sure. Although only if I were involved after the fact.”
Describe your experience using the assessment checklist in real-time	“The first time was a little challenging, but I had no issues after.” “I would have liked a few more minutes beforehand to familiarize myself with the checklist.” “I thought the checklist was well organized and I was able to anticipate what the learners would do next and score appropriately.”
Do you feel the checklist accurately assesses the pediatric clerkship students during their infant respiratory distress simulation-based learning module?	“Yes. I found it very thorough and representative of what we are teaching them.” “Yes. It targets all of the major themes as well as the objectives.”

## Discussion

Measuring clinical performance is paramount to developing and assessing effective educational interventions and learner competency. This project describes a robust, thorough, and systematic approach to designing a checklist for the simulated clinical performance of infant respiratory distress. By using approach, we designed a checklist with validity evidence that accurately measures learner performance, with strong inter-rater reliability for a specific SBL scenario.

This work builds on previous work in multiple ways. First, the project supports the effectiveness of this previously described methodology of the five-step modified Delphi checklist development process for clinical performance [7]. Second, in recognizing the importance of learning objectives, our group included learning objectives in the checklist development process. Third, we added a qualitative component in an attempt to evaluate the checklist development process itself. We found that the participants did not find the process overly time consuming, that the assessment tool measured our specific metrics, and was easy to use in real-time.

There are numerous publications utilizing various assessment tool development methods. Most relevant to this report is the work by Schmutz et al. describing the modified Delphi method that formed the basis for our approach [7]. By adding a final review round after the pilot phase, as compared to other methods, Schmutz et al. were able to avoid rater bias based on personal opinions and experiences [7, 14-16]. Rater bias in our study could be overcome with a larger reviewer group but that would likely add complexity and time to the process. We feel the small panel size added to the efficiency and ease of use of the development process. Without the final Delphi review round, a larger expert group would have likely been needed.

One prior study reported their modified Delphi process was time consuming [7]. We found the opposite. Possible reasons for this include more robust development of our initial draft checklist (prior to our first modified Delphi round), therefore requiring less revision and refinement, intimate involvement of our panel as faculty in the infant respiratory distress SBL module, and previous experience of three experts with a similar modified Delphi experience.

There are a number of recent publications using a form of the Delphi method including but not limited to teamwork and communication in trauma management situations, neonatal intubation, and assessment of milestones for emergency medicine residents [17-19]. These efforts speak to the generalizability of the modified Delphi process. Because developed checklists are specific to a given situation and/or learner group, generalization of checklists themselves can be challenging. By adding the ease of use of qualitative data, we hope clinical educators and researchers will find it worthwhile to use this five-step process to develop their own specific checklists to better assess their interventions that require evaluation.

Limitations

The first limitation of our work is feasibility of the modified Delphi process. Other methods, such as global rating scales, are less time intensive but are not as thorough and complete for measuring clinical performance. We encourage educators and researchers to consider assessment needs prior to selecting a specific development process.

A second limitation is that the primary author was involved in all phases of the process, which could have led to bias. We minimized this effect by including experts not involved in the development process for the pilot phase and the implementation/inter-rater reliability testing phase.

A third limitation is the small group size of experts from the same institution used in this process. We made a purposeful decision to include experts familiar with the educational intervention we were assessing to add efficiency and specificity to the process. An outside expert may have provided insight or edits to the checklist and further minimized personal bias from the reviewers. Of note, the specificity of our assessment tool, as well as the composition of subject experts, makes this tool difficult to generalize beyond the simulated scenario described. More importantly, we feel the development process itself is very generalizable.

A final limitation is the lack of deeper psychometric evaluation of the checklist. In the future, our group plans to use this checklist to evaluate our educational intervention and publish the curriculum, including further psychometric analysis. This additional process will allow others to implement the curriculum with an evaluation strategy in place and add construct validity.

## Conclusions

Determining effective ways to measure clinical performance is important not only for learner education but patient safety and outcomes as well. We have described a comprehensive and integrative approach to measuring simulated clinical performance. We have also shown that this process is less time consuming and less resource intense than other checklist development methods as well as previous modified Delphi work. There is no "perfect method" to assessment. One must consider the purpose for assessment as well as time/resource availability and potential outcome of the assessment. By highlighting the ease of use of this development process, we hope others will add this modified Delphi approach to their toolbox to enhance their own assessment strategies.
